# Editorial: Wound repair: establishment and development of a new discipline in China

**DOI:** 10.3389/fsurg.2023.1207709

**Published:** 2023-05-16

**Authors:** Haihong Li, Biao Cheng, Cuiping Zhang

**Affiliations:** ^1^ Department of Wound Repair, Southern University of Science and Technology Hospital, Shenzhen, China; ^2^ Department of Plastic Surgery, General Hospital of Southern Theater Command, PLA, Guangzhou, China; ^3^ Research Center for Tissue Repair and Regeneration Affiliated to the Medical Innovation Research Department, Fourth Medical Center of PLA General Hospital, Beijing, China

**Keywords:** wound repair, wound healing, wounds, discipline, management

**Editorial on the Research Topic** Wound repair: establishment and development of a new discipline in China

Chronic wounds have become a global concern as the population ages and the incidence of obesity, diabetes and vascular disease increases (1). It is estimated that in some countries, approximately 1%–2%, and even up to 10% of the population will experience chronic wounds during their lifetime (2, 3). In the United States, wounds affect approximately 8.2 million of Medicare beneficiaries (4); in Europe, an estimated 1.5–2 million people suffer from acute or chronic wounds (5); in China, chronic wounds affect approximately 50 million people annually (2). Wounds, especially chronic wounds, not only affect the quality of life of patients, but also bring huge physical, psychological and economic burdens to patients and their families, as well as the medical system and society (1, 6, 7).

There are many types of chronic wounds, including pressure ulcers, diabetic ulcers, venous ulcers, arterial ulcers, traumatic ulcers, burn residual wounds, radiation ulcers, and iatrogenic ulcers (8, 9). Due to the complex etiology and clinical manifestations of chronic wounds, wound patients are admitted to multiple clinical departments such as dermatology, endocrinology, orthopedics, burn and plastic surgery, general surgery, trauma surgery, and emergency (9). However, wound management approaches, costs, and outcomes vary widely across departments. To standardize wound management, some hospitals have established wound healing centers (WHCs).

The earliest WHC in Denmark was established in 1996, and in China in 1999 (9, 10). Wound treatment has made great progress in the past few decades, and many innovative technologies such as modern debridement, growth factor, bioengineered skin, vacuum sealing drainage (VSD), and new medical dressings have been applied clinically. The experience of the European Wound Management Association, the World Union of Wound Healing Societies, the Danish Wound Healing Society, and the Chinese Wound Healing Team shows that WHCs focused on all types of wounds provides the most effective treatment and care for wound patients, and that the best way to optimize wound treatment is through professional education and training (8–12).

Over the past 10 years, more and more WHCs have been established in the Tertiary-Hospitals in China. Although wound patients receive better treatment and care at the WHCs, practitioners have no wound management guidelines to follow. With the in-depth understanding of wound management, a new Third-level Discipline of Clinical Medicine—the Discipline of Wound Repair has been put on the agenda (9). In March 2019, Professor Xiaobing Fu, as an advocate, proposed to the Chinese National Health Commission to establish the Discipline of Wound Repair, which was approved in November 2019. The establishment of the Discipline of Wound Repair has epoch-making significance for promoting the construction of the discipline system of Wound Repair, cultivating professionals, accelerating the process of wound standardization, and improving the overall level of wound repair in China.

As an emerging discipline, the discipline of wound repair is not widely known, including by some medical practitioners. A comprehensive understanding of the etiology, prevention, diagnosis, and treatment of different wounds, as well as the commonalities or characteristics of different wounds, is urgently needed by all relevant practitioners. The topic of “Wound Repair: The Establishment and Development of a New Discipline in China” aims to introduce the development of the Discipline of Wound Repair, the research progress of wound repair, and all aspects related to wound repair in China.

A total of 15 manuscripts were accepted in the topic, including 11 original articles, 2 reviews, 1 opinion piece, and 1 case report, all from China. Huang et al. review “The establishment and development of wound repair discipline in China” in detail. Zhao et al. introduce a wound assessment tool for laypeople-doctor communication in pressure ulcer prevention. Many treatment methods, such as Masquelet technique combined with dermoplasty for human tendon-exposed wounds, surgical amputation for human diabetic foot ulcers, local autologous platelet rich plasma (PRP) injection combined with platelet rich fibrin (PRF) filling for human refractory wounds, double-layer artificial dermis for human nail bed reconstruction, resorbable membrane for closure of human palatal fistulas, integrated surgical wound management for human diabetic foot wounds, extracellular vesicles (EVs) for mouse diabetic wounds, and bacterial cellulose membrane combined with mesenchymal stem cells (MSCs) for mouse acute full-thickness skin defect, have been explored. RNA-sequencing studies have shown that circRNAs, miRNAs, and their network are involved in the development of refractory diabetic wounds. Gene expression microarray data have revealed that ferroportin (SLC40A1) and alanine transaminase 2 (GPT2) are associated with overall survival in burn patients and can be used as biomarkers to predict the prognosis of burn patients. The bibliometric and visualization study on the global trends of chronic wound-related high-throughput sequencing shows that “prevalence”, “gene expression”, “inflammation” and “infection” have become recent hot spots. However, despite tremendous advances in clinical and basic research on wounds, common standards and guidelines for wound management are still missing (9). By focusing on wound healing issues, we hope that this research topic will play a role in advancing the Discipline of Wound Healing.

## References

[1] Sen CK. Human wound and its burden: updated 2020 compendium of estimates. Adv Wound Care. (2021) 10:281–92. 10.1089/wound.2021.0026 PMC8024242 33733885

[2] Shen C. 337 New technologies for precision repair of refractory wounds post burn and trauma. J Burn Care Res. (2019) 40:S145–S145. 10.1093/jbcr/irz013.248

[3] Järbrink K Ni G Sönnergren H Schmidtchen A Pang C Bajpai R Prevalence and incidence of chronic wounds and related complications: a protocol for a systematic review. Syst Rev. (2016) 5:152. 10.1186/s13643-016-0329-y 27609108 PMC5017042

[4] Nussbaum SR Carter MJ Fife CE DaVanzo J Haught R Nusgart M An economic evaluation of the impact, cost, and medicare policy implications of chronic nonhealing wounds. Value Health. (2018) 21:27–32. 10.1016/j.jval.2017.07.007 29304937

[5] Lindholm C Searle R. Wound management for the 21st century: combining effectiveness and efficiency. Int Wound J. (2016) 13(Suppl 2):5–15. 10.1111/iwj.12623 27460943 PMC7949725

[6] Jiang Y Huang S Fu X Liu H Ran X Lu S Epidemiology of chronic cutaneous wounds in China. Wound Repair Regen. (2011) 19:181–8. 10.1111/j.1524-475X.2010.00666.x 21362085

[7] Järbrink K Ni G Sönnergren H Schmidtchen A Pang C Bajpai R The humanistic and economic burden of chronic wounds: a protocol for a systematic review. Syst Rev. (2017) 6:15. 10.1186/s13643-016-0400-8 28118847 PMC5259833

[8] Sholar AD Wong LK Culpepper JW Sargent LA. The specialized wound care center: a 7-year experience at a tertiary care hospital. Ann Plast Surg. (2007) 58:279–84. 10.1097/01.sap.0000248116.28131.94 17471132

[9] Jiang Y Xia L Jia L Fu X. Survey of wound-healing centers and wound care units in China. Int J Low Extrem Wounds. (2016) 15:274–9. 10.1177/1534734614568667 25724595

[10] Gottrup F. Organization of wound healing services: the Danish experience and the importance of surgery. Wound Repair Regen. (2003) 11:452–7. 10.1046/j.1524-475X.2003.11609.x 14617285

[11] Gottrup F. Optimizing wound treatment through health care structuring and professional education. Wound Repair Regen. (2004) 12:129–33. 10.1111/j.1067-1927.2004.012204.x 15086763

[12] Jiang Y Lu S Wen B Fu X. Improving would healing ability by training: experiences of China. Int J Low Extrem Wounds. (2018) 17:190–4. 10.1177/1534734618796589 30178693

